# Relations between Air Quality and Covid-19 Lockdown Measures in Valencia, Spain

**DOI:** 10.3390/ijerph18052296

**Published:** 2021-02-26

**Authors:** Gabriele Donzelli, Lorenzo Cioni, Mariagrazia Cancellieri, Agustin Llopis-Morales, María Morales-Suárez-Varela

**Affiliations:** 1 Department of Preventive Medicine and Public Health, Food Sciences, Toxicology, and Legal Medicine, School of Pharmacy, University of Valencia, Avenida Vicente Andres Estellés s/n, Burjassot, 46100 Valencia, Spain; allomo3@alumni.uv.es (A.L.-M.); Maria.M.Morales@uv.es (M.M.-S.-V.); 2 Department of Health Sciences, University of Florence, Viale GB Morgagni 48, 50134 Florence, Italy; 3 Scuola Normale Superiore, Piazza dei Cavalieri 7, 56126 Pisa, Italy; lorenzo.cioni@sns.it; 4 Hygiene and Public Health Unit, Department of Public Health, AUSL Imola, Viale Giovanni Amendola 2, 40026 Bologna, Italy; m.cancellieri@ausl.imola.bo.it; 5 Biomedical Research Consortium in Epidemiology and Public Health Network (CIBERESP), Avenida Monforte de Lemos, 3-5. Pabellón 11. Planta 0, 28029 Madrid, Spain

**Keywords:** Covid-19, lockdown, air pollution, particulate matter (pm), nitrogen oxides, ozone

## Abstract

The set of measures to contain the diffusion of COVID-19 instituted by the European governments gave an unparalleled opportunity to improve our understanding of the transport and industrial sectors’ contribution to urban air pollution. The purpose of this study was to assess the impacts of the lockdown measures on air quality and pollutant emissions in Valencia, Spain. For this reason, we determined if there was a significant difference in the concentration levels of different particulate matter (PM) sizes, PM_10_, PM_2.5_, and NO_x_, NO_2_, NO, and O_3,_ between the period of restrictions in 2020 and the same period in 2019. Our findings indicated that PM pollutant levels during the lockdown period were significantly different from the same period of the previous year, even if there is variability in the different local areas. The highest variations reduction in the PM_10_ and PM_2.5_ levels were observed for the València Centre, València Avd Francia, and València Pista de Silla (all of the urban traffic type) in which there was a reduction of 58%–42%, 56%–53%, and 60%–41% respectively. Moreover, consistent with recent studies, we observed a significant reduction in nitric oxide levels in all the air monitoring stations. In all seven monitoring stations, it was observed, in 2020, NO_x_, NO_2_, and NO concentrations decreased by 48.5%–49.8%–46.2%, 62.1%–67.4%–45.7%, 37.4%–35.7%–35.3%, 60.7%–67.7%–47.1%, 65.5%–65.8%–63.5%, 60.0%–64.5%–41.3%, and 60.4%–61.6%–52.5%, respectively. Lastly, overall O_3_ levels decreased during the lockdown period, although this phenomenon was more closely related to weather conditions. Overall, no significant differences were observed between the meteorological conditions in 2019 and 2020. Our findings suggest that further studies on the effect of human activities on air quality are needed and encourage the adoption of a holistic approach to improve urban air quality.

## 1. Introduction

Air pollution is a significant cause of premature death and disease in Europe. The latest estimates made by the European Environment Agency (EEA) show that health impacts attributable to fine particulate matter (PM_2.5_) exposure is responsible for about 412,000 premature deaths and that NO_2_ and O_3_ are respectively responsible for about 71,000 and 15,100 premature deaths per year. Spain is one of the countries with the most people exposed to harmful levels of air pollution levels, with 24,100 premature deaths due to PM_2.5_ exposure, 7700 to NO_2_ exposure, and 1500 to O_3_ exposure [1].

Road transport is one of the most important sources of emissions of air pollutants in towns and cities, and it is responsible for almost a quarter of greenhouse gas emissions in Europe [2]. More than half of Spain’s population is exposed to excessive levels of particulate matter, nitrogen dioxide, and ozone. Ecologists in Action (https://www.ecologistasenaccion.org/) (accesed on 14 January 2021), a known environmental organization, estimates that 17.5 million of Spain’s population was affected by the poor air quality in 2017 [3].

Meteorological parameters play a significant role in determining air pollutants concentrations [4]. Decreases in PM concentrations were observed with increases in precipitation rate, wind speed, and temperature [5]. Sun angle, wind speed, temperature, and nitrogen oxide (NO_x_) emissions determine the NO_2_ concentrations in the air. Changes in atmospheric conditions between years can cause column NO_2_ differences of about 15% over monthly timescales [6]. O_3_ levels rise with high temperatures and decrease with high relative humidity [7].

Although the human, social and economic impacts of the outbreak have hit all EU countries [8,9], the lockdown measures implemented by the European governments gave an unparalleled opportunity to improve our understanding of how human activities contribute to air pollution in towns and cities. The adoption of mandatory measures has indeed significantly reduced transport and industry-related emissions. Transport was one of the most severely affected sectors by the pandemic, along with sectors such as tourism and hospitality. Tourism largely ceased in March 2020 due to international travel bans affecting over 90% of the world population and wide-spread restrictions on public gatherings and community mobility [10]. Regarding the aviation sector, a recent article reported that the reduction in the number of flights was over 89% for the EU region [11]. Also, it is estimated that restrictive measures implemented by Italian authorities during the lockdown resulted in a reduction of ~64.6% in private vehicle use in Rome [12]. The Air quality in Europe—2020 report showed that NO_2_ and PM_10_ concentrations generally reduced across Europe as a result of lockdown measures and independently of the meteorological conditions [13].

On 14 March 2020, Spain’s prime minister declared a nationwide state of emergency to reduce the diffusion of the coronavirus [14]. Spanish government imposed a lockdown, reducing all unnecessary movements of the people across the country, except for buying food or medicines or going to work or hospital. After the severe lockdown, Spain’s Government implemented a three-phase de-escalation plan, which allowed the return to the new normality [15]. 

The purpose of this study was to assess the impacts of lockdown measures on air quality and pollutant emissions in Valencia, Spain. In fact, to date, there is no study that has completely evaluated the changes in air quality during the pandemic in Valencia, and therefore, we think that our study can contribute to the increasing knowledge about the urban area of the city. For this reason, we investigated if there was a significant difference in the concentration levels of PM_10_, PM_2.5_, NO_x_, NO_2_, NO, and O_3_ between the period of the restrictions in 2020 and the same period in 2019.

## 2. Methods

### 2.1. Area of the Study

**Figure 1:**
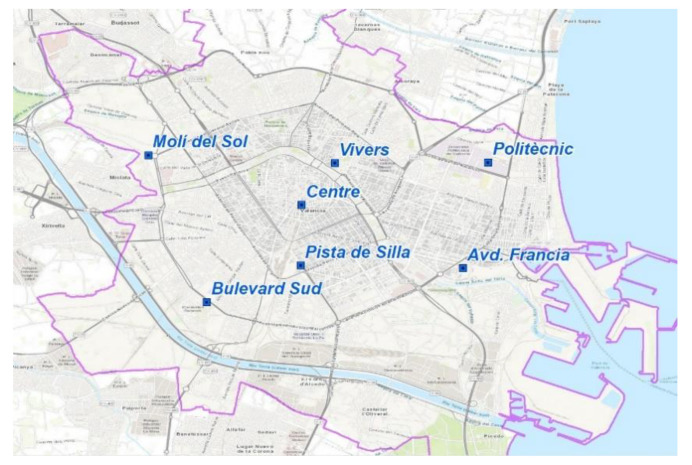
Map of air monitoring stations included in the study.

Valencia (Figure 1) is a city with around 800,000 inhabitants, and it is the center of an expansive metropolitan area reaching a million and a half inhabitants. The population of Valencia represents 18% of the Comunidad Valenciana population, and Valencia is the third-largest city in Spain after Madrid (which has about 3,000,000 inhabitants) and Barcelona (which has approximately 1,600,000 inhabitants).

### 2.2. Air Pollution and Meteorological Data Collection

Air pollution data were collected from the Generalitat Valenciana website (http://www.agroambient.gva.es/es/web/calidad-ambiental/datos-historicos) (accesed on 14 January 2021). The analyses include measures of the daily averages of six pollutants (PM_10_, PM_2.5_, NO_2_, NO, NO_x_, and O_3_) ranging over a period from 1 January 2019, to 30 September 2020, with occasional gaps because of unavailable data. We named all these months the sampling period. More specifically, we considered the data from 1 January to 30 September of 2019 and the data from 1 January to 30 September of 2020 to perform the statistical analyses over the two corresponding time periods. We named these two timeframes the common periods.

Data were collected from all the air quality monitoring stations of the city of Valencia. For each air monitoring station, we indicated in Table 1 the type of the measured pollutants. The type of the measured pollutants in each air monitoring station varied according to Table 1. Data verification and validation were provided by the Generalitat Valenciana through the monitoring of the instrumental performance and the application of quality control procedures. The sensors of the Valencian Network of Surveillance and Control stations performed air analysis in real-time. Each air quality station is equipped with automatic continuous measurement monitors as established in the EU Directive 2008/50/EC. For the determination of pollutants, we used equipment based on the official methods included in Annex VII of the R.D. 102/2011, of 28 January, relative to the improvement of air quality, modified by Royal Decree 39/2017, of 27 January. It is possible to find more specific information about the air quality network of the website of the Generalitat Valenciana [16].

For each pollutant, from 1 January 2019 to 30 September 2020, we considered the two common periods and divided them further into the following subperiods to be pairwise compared:

- the period from 1 January to 14 March 2020 (the so-called Pre-lockdown or Normality 1 period) to be compared with the period from 1 January to 14 March 2019;
- the period from 15 March to 17 May 2020 (the so-called Lockdown or Phase 0 period) to be compared with the period from 15 March to 17 May 2019;
- the period from 18 May to 31 May 2020 (the so-called Phase 1 period) to be compared with the period from 18 May to 31 May 2019;
- the period from 1 June to 14 June 2020 (the so-called Phase 2 period) to be compared with the period from 1 June to 14 June 2019;
- the period from 15 June to 20 June 2020 (the so-called Phase 3 period) to be compared with the period from 15 June to 20 June 2019;
- the period from 21 June to 30 September 2020 (the so-called Post-lockdown or Normality 2 period) to be compared with the period from 21 June to 30 September 2019.

During the Lockdown period, citizens had to remain in their primary residences except to purchase food and medicines, work, or attend an emergency. Only essential businesses, including grocery stores and food trade, pharmacies, and other industries, were authorized to be open. While other non-essential businesses such as retail shops, bars, restaurants, and commercial companies, had to close [17]. After the lockdown, businesses in some non-essential sectors, including construction and industry, were gradually allowed to return to work. The lockdown de-escalation plan consisted of three phases. The government slowly eased restrictions, and people resumed some of their everyday activities, reaching a so-called “new normality” [18]. For example, the social gathering limit was increased from 10 people to 15 when Phase 1 moved to Phase 2. Moreover, in Phase 2, there were no restrictions on outdoor activity. In Phase 3, bars were allowed to reopen at 50% capacity, shoppers could enter establishments up to 50% capacity, and social gatherings of up to 20 were allowed.

Descriptive statistics (mean and standard deviation) were used to summarize the level of pollution for each subperiod identified. A paired *t*-test was used to determine if there was a significant difference between the mean concentrations of the above-mentioned subperiods in 2019 and 2020. A *p*-value < 0.05 was chosen to reject the null hypothesis, which assumes that the true mean difference between the paired samples was zero. It was assumed that the observations were independent of one another, and it was verified that data were normally distributed. It was also examined the relative percentage variation ((value 2020 − value 2019)/value 2019) × 100 for each subperiods. All statistical calculations were performed using XLSTAT software (Addinsoft, Paris, France) (https://www.xlstat.com/en/) (accessed on 14 January 2021).

For this analysis, we also downloaded the data on wind speed, rainfall, relative humidity, temperature, atmospheric pressure, and solar irradiance for the entire period from the Generalitat Valenciana website (http://www.agroambient.gva.es/es/web/calidad-ambiental/datos-historicos) (accessed on 14 January 2021).

Indeed, we downloaded the meteorological data (under the form of average daily values) as measured at the specialized València-Conselleria Meteo station (the only station in València with meteorological data both for 2019 and 2020): average daily air temperature (as Celsius degrees), average daily air humidity (as a percentage value), maximum wind speed (as m/s), average daily wind speed (as m/s), wind direction (as degrees), average daily sea-level atmospheric pressure (as mbar), solar irradiance (W/m^2^), and daily mean precipitation (as l/m^2^).

On such data, we performed a series of paired *t*-tests (*p*-value < 0.05) in order to determine if there was a significant difference between the mean concentrations of the above-mentioned subperiods in 2019 and 2020. In a similar way to what was done in the analysis of pollutants, a paired *t*-test was used to determine if there was a significant difference (*p*-value < 0.05) between the meteorological conditions of the above-mentioned subperiods in 2019 and 2020.

## 3. Results and Discussion

### 3.1. Particulate Matter

PM_10_ and PM_2.5_ levels during the normality period are not statistically significantly different from the same period in 2019 in most of the air monitoring stations located in the city of Valencia (Table 2). The only exceptions are València Centre, València Pista de Silla and València Vivers for PM_10_, and València Avd Francia for PM_2.5_. Except for València Vivers, where was observed an increase in PM_10_ in 2020, for the other three air monitoring stations was observed a decrease in PM in 2020.

As shown in Table 2, COVID-19 lockdown measures induced a notable reduction in the PM_10_ and PM_2.5_ levels in Valencia but for València Vivers (PM_10_, urban background type) and València Politècnic (PM_2.5_, suburban background type). The effect of the COVID-19 outbreak on particulate matter levels has already been investigated in numerous studies, which confirmed a substantial reduction in PM levels [19,20,21,22,23,24,25,26,27]. Data were obtained from the environmental agencies, and periods with different levels of mobility restrictions were compared. Most of these studies analyzed both PM_10_ and PM2.5 concentrations and considered the meteorological data. For example, in a recent study on global environmental pollution, an important decrease in PM concentration levels was observed in most of the considered cities, in which PM_10_ and PM_2.5_ levels were reduced by 24%–47% and 20%–34%, respectively [28].

PM_10_ concentrations were significantly decreased during the lockdown in three Spanish cities, Barcelona, Valencia, and Sevilla [29]. Regarding the city of Valencia, which was the subject of that study, only one air monitoring station was considered, and therefore we believe that it cannot be considered as representative for the whole city. For this reason, we think that our study can contribute to the increasing knowledge of the urban area of the city of Valencia. In this regard, we observed notable variations in the reductions of PM_10_ and PM_2.5_ levels, with the highest values for the València Centre, València Avd Francia, and València Pista de Silla (all of the urban traffic type) in which we observed a reduction of 58%–42%, 56%–53%, and 60%–41% respectively. The variation of the environmental response, which was observed in the different air monitoring stations, is probably due to the dominant source of emission in each area [30]. This suggests that it is important to consider any additional PM sources and encourage the adoption of a holistic approach to improve urban air quality [31].

### 3.2. Nitrogen Oxides

The burning of fuel of cars, power plants, trucks and buses, and off-road equipment are the main sources of NO_x_ in the air. NO_x_ reacts with other substances to produce PM and O_3_. It is damaging because it can be inhaled and affect the respiratory system. NO_x_, NO_2_, and NO concentrations significantly decrease (*p*-value < 0.05) during the lockdown period compared to the same period of 2019 in all the air monitoring stations included in this study (Table 3). These findings show that the reduction in traffic and industrial emissions has direct effects on reducing nitric oxide concentrations and should be used as evidence for implementing public policies. More specifically, they should motivate and allow the adoption of urban mobility plans for the improvement of the air quality in urban environments [32].

In details, NO_x_, NO_2_, and NO concentrations in 2020 decreased on average by 48.5%–49.8%–46.2%, 62.1%–67.4%–45.7%, 37.4%–35.7%–35.3%, 60.7%–67.7%–47.1%, 65.5%–65.8%–63.5%, 60.0%–64.5%–41.3%, and 60.4%–61.6%–52.5%, respectively, at the València Centre, València Avd Francia, València Bulevard Sud, València Pista de Silla (all the urban traffic type), València Molí del Sol (suburban traffic type), València Politècnic (suburban background type), and València Vivers (urban background type) air monitoring stations. Our results were consistent with recent studies. Strong reductions in nitrogen dioxide concentrations over several major cities across Europe, including Paris, Madrid, and Rome, were shown by the Copernicus Sentinel-5P satellite [33]. A decrease of up to 54.3% in NO_2_ concentrations was observed in São Paulo state, Brazil [24], as well as in Milan, Italy, where NO_x_ and NO_2_ drastically dropped [20]. A decrease in NO_2_ levels was also observed in different regions of India [23], in Rio de Janeiro, Brazil [22], in the megacity of Delhi, India [21], in the Yangtze River Delta Region [25], and 44 cities in northern China [26].

NO_x_, NO_2_, and NO concentrations were significantly lower in 2020 compared with 2019, even in the normality periods (Table 3). These variations are definitely higher when lockdown measures were applied and more than doubled in some cases. These results confirm the importance of vehicular traffic in determining the concentrations of nitrogen oxides and were consistent with recent studies that identified road traffic as one of the most important sources of nitrogen dioxide [34] and showed a direct relationship between traffic and NO_2_ (see for instance [27,28,29]).

### 3.3. Ozone

In the lower troposphere, O_3_ is a damaging pollutant that is produced by chemical reactions between volatile organic compounds and nitrogen oxides in the presence of heat and sunlight. Tropospheric O_3_ production is largely dependent on the concentration of NO_x_ [35,36,37,38] in photochemical reactions. Table 4 shows the ozone levels across the six periods. Overall, we can observe a decrease in O_3_ concentrations, which is likely due to the drop in traffic volumes compared with 2019, apart from Phase 1. This difference may be explained by the significant increase in temperature in Phase 1 and an increase in solar irradiance, which is strongly correlated with the ozone concentrations [39]. Furthermore, this increase could also be due to the significant reduction in NO_2_ concentrations, as reported by several articles [40,41,42].

### 3.4. Meteorological Data

For what concerns the meteorological data (average daily values) as measured at the specialized València-Conselleria Meteo station in 2019 and 2020 we found:

- no statistically significant (*p*-value < 0.05) differences for maximum wind speed, wind direction, and daily mean precipitations;
- negligible differences for average daily wind speed, average daily sea-level atmospheric pressure, and solar irradiance;
- somewhat greater differences for average daily air temperature and average daily air humidity but not so great as to let us suspect of any impact of such differences on the distributions of the analyzed pollutants.

From the above, we can conclude that the two years 2019 and 2020 can be considered as highly similar from a meteorological point of view.

## 4. Conclusions

This article was aimed at assessing how the lockdown measures implemented by the Spanish government to contain the COVID-19 outbreak impacted the levels of air pollutants in Valencia. The reduction in all unnecessary movements of the people across the country except for buying food or medicine or going to work or hospital gave an unparalleled opportunity to evaluate the impact of human activities on urban air quality. In particular, the lockdown measures that were adopted significantly reduced human activities, like car use and industrial production. We aimed to evaluate the impact of the lockdown measures on the levels of PM_10_, PM_2.5_, NO_x_, NO_2_, NO, and O_3_, using data collected by the Generalitat Valenciana. For reaching this goal, we choose to compare air pollutant concentrations in 2020 with the previous year since we noticed in the literature similar studies which used this methodology. Our findings showed that lockdown measures were accompanied by a significant decrease in PM concentrations, even if there was variability in various areas of the city. The highest variations reduction in the PM_10_ and PM_2.5_ levels were observed for the València Centre, València Avd Francia, and València Pista de Silla (all of the urban traffic type) in which there was a reduction of 58%–42%, 56%–53%, and 60%–41% respectively. Our findings are consistent with recent studies on this subject, even if we have to take into account that we can observe variations in the environmental response due to the prevalent source of emission and the weather conditions. Moreover, we found a statistically significant decrease in NO_x_, NO_2_, and NO concentrations at all seven air monitoring stations by 48.5%–49.8%–46.2%, 62.1%–67.4%–45.7%, 37.4%–35.7%–35.3%, 60.7%–67.7%–47.1%, 65.5%–65.8%–63.5%, 60.0%–64.5%–41.3%, and 60.4%–61.6%–52.5%, respectively. Lastly, we also observed a significant decrease in O_3_ levels during the lockdown period. Overall, no significant differences were observed among the meteorological conditions in 2019 and 2020 (data not shown). In general, we should take into account that the more traffic emissions are reduced, the more residential emissions increase. Some first estimates in France showed that an increase of 20% of residential emissions could be observed. In conclusion, we believe that our results should be taken into account by policymakers to implement effective policies to contrast air pollution and to place human health at the center of urban planning.

## Figures and Tables

**Table 1:** Characteristics of the air monitoring stations included in the present study.

Monitoring Station	Address	Area Type	Pollutants					
			PM_10_	PM_2.5_	NO_x_	NO_2_	NO	O_3_
*València Centre*	*Plaça de* *l’Ajuntamento*	*urban* *traffic*	✓	✓	✓	✓	✓	
*València Avd Francia*	*Avenida de Francia*	*urban* *traffic*	✓	✓	✓	✓	✓	
*València Bulevard Sud*	*Bulevar Sur s/n (Parking* *cementerio* *de Valencia)*	*urban* *traffic*	✓		✓	✓	✓	
*València Pista de Silla*	*C/Filipinas s/n frente nº 37*	*urban* *traffic*	✓	✓	✓	✓	✓	✓
València Molí del Sol	Av. Pio Baroja, s/n	suburban traffic	✓	✓	✓	✓	✓	✓
València Vivers	Jardines de Viveros	urban background	✓	✓	✓	✓	✓	✓
València Politècnic	Camino de Vera, s/n	suburban background	✓	✓	✓	✓	✓	✓

**Table 2:** PM_10_ and PM_2.5_ mean and standard deviation (σ) are expressed in μg∙m^−3^. A green color denotes a significant (*p*-value < 0.05) decrease, whereas an orange color denotes a significant increase between 2019 and 2020.

PM_10_	Normality 1		Lockdown		Phase 1		Phase 2		Phase 3		Normality 2	
	2019 µ(σ)	2020 µ(σ)	2019 µ(σ)	2020 µ(σ)	2019 µ(σ)	2020 µ(σ)	2019 µ(σ)	2020 µ(σ)	2019 µ(σ)	2020 µ(σ)	2019 µ(σ)	2020 µ(σ)
*València Centre*	39.1 (14.0)	31.7 (16.0)	26.5 (10.2)	11.2 (6.1)	19.8 (5.0)	12.6 (8.3)	22.5 (6.4)	12.3 (6.9)	21.0 (1.8)	13.2 (2.6)	17.3 (6.0)	12.7 (9.9)
*València Avd Francia*	31.8 (13.7)	25.0 (28.2)	21.3 (8.9)	9.3 (3.9)	15.6 (5.0)	11.4 (4.4)	21.3 (7.4)	12.4 (7.0)	22.9 (2.7)	7.4 (2.4)	21.3 (8.0)	9.7 (3.4)
*València Bulevard Sud*	36.7 (11.8)	43.7 (24.0)	27.4 (5.7)	21.0 (4.9)	24.7 (2.1)	20.0 (7.9)	n.a.	n.a.	n.a.	n.a.	28.3 (4.5)	25.5 (5.7)
*València Pista de Silla*	34.5 (11.4)	24.6 (15.4)	21.4 (10.8)	8.6 (4.5)	15.1 (5.0)	13.9 (6.1)	18.3 (7.4)	16.0 (5.7)	20.7 (2.3)	15.8 (3.7)	23.4 (8.4)	12.3 (4.2)
València Molí del Sol	27.3 (12.4)	30.3 (17.3)	21.0 (8.6)	14.0 (8.6)	15.8 (5.2)	12.9 (2.9)	17.7 (5.8)	10.4 (3.1)	25.7 (7.2)	12.2 (1.3)	24.3 (9.3)	10.1 (5.0)
València Vivers	27.0 (10.4)	38.6 (21.4)	19.6 (7.2)	17.0 (4.3)	15.7 (3.0)	20.7 (4.1)	25.8 (7.5)	23.8 (6.2)	n.a.	n.a.	23.8 (6.8)	19.8 (4.0)
València Politècnic	26.2 (11.4)	22.1 (12.2)	19.6 (9.7)	12.4 (5.6)	17.9 (4.9)	13.9 (3.6)	21.0 (6.9)	9.7 (3.4)	24.7 (2.8)	7.2 (1.5)	17.8 (7.2)	14.3 (4.2)
**PM_2.5_**	**Normality 1**		**Lockdown**		**Phase 1**		**Phase 2**		**Phase 3**		**Normality 2**	
	**2019** **µ(σ)**	**2020** **µ(σ)**	**2019** **µ(σ)**	**2020** **µ(σ)**	**2019** **µ(σ)**	**2020** **µ(σ)**	**2019** **µ(σ)**	**2020** **µ(σ)**	**2019** **µ(σ)**	**2020** **µ(σ)**	**2019** **µ(σ)**	**2020** **µ(σ)**
*València Centre*	22.6 (11.1)	21.5 (11.6)	15.9 (7.5)	9.3 (9.0)	10.8 (3.1)	7.0 (5.3)	10.9 (3.1)	6.5 (4.8)	13.2 (3.3)	7.7 (1.0)	10.7 (3.7)	6.7 (3.3)
*València Avd Francia*	19.8 (10.1)	14.5 (11.3)	13.9 (6.6)	6.5 (3.0)	10.2 (3.6)	5.9 (1.6)	10.4 (3.0)	5.1 (1.4)	14.5 (3.5)	4.5 (0.5)	12.8 (4.7)	4.8 (2.3)
*València Pista de Silla*	16.1 (8.6)	14.7 (9.1)	11.9 (5.9)	7.0 (4.5)	7.7 (3.1)	6.6 (2.9)	8.2 (2.8)	7.2 (3.2)	12.2 (3.3)	8.3 (1.0)	13.2 (5.1)	7.7 (3.2)
València Molí del Sol	23.8 (12.2)	26.7 (15.6)	18.3 (8.2)	12.8 (8.2)	13.9 (4.7)	11.6 (2.7)	14.6 (4.4)	9.1 (2.8)	22.2 (7.1)	10.8 (1.2)	20.6 (8.2)	9.3 (4.7)
València Vivers	16.9 (6.6)	19.0 (8.0)	13.0 (6.0)	9.1 (3.2)	11.2 (2.5)	12.1 (2.4)	14.4 (3.4)	11.4 (3.6)	16.8 (2.6)	12.5 (0.6)	15.0 (3.9)	11.2 (2.9)
València Politècnic	17.0 (9.3)	17.7 (10.9)	12.6 (6.6)	11.0 (5.8)	11.4 (3.8)	10.1 (2.8)	11.4 (3.2)	6.3 (2.5)	16.2 (4.6)	5.2 (1.0)	12.2 (4.7)	9.9 (3.5)

**Table 3:** NO_x_, NO_2_, and NO means and standard deviations are expressed in μg∙m^−3^. Green color denotes a significant (*p*-value < 0.05) decrease between 2019 and 2020.

NO_x_	Normality 1		Lockdown		Phase 1		Phase 2		Phase 3		Normality 2	
	2019 µ(σ)	2020 µ(σ)	2019 µ(σ)	2020 µ(σ)	2019 µ(σ)	2020 µ(σ)	2019 µ(σ)	2020 µ(σ)	2019 µ(σ)	2020 µ(σ)	2019 µ(σ)	2020 µ(σ)
*València Centre*	94.0 (35.8)	73.0 (32.6)	50.0 (18.4)	25.7 (7.9)	48.9 (13.5)	25.6 (7.3)	42.3 (7.4)	26.9 (4.5)	32.5 (5.5)	30.8 (4.3)	50.7 (16.6)	43.4 (11.4)
*València Avd Francia*	60.2 (33.2)	40.2 (23.6)	25.8 (12.9)	9.8 (4.6)	24.3 (6.9)	14.6 (6.2)	16.4 (4.4)	12.4 (3.7)	15.4 (4.8)	10.4 (2.1)	18.3 (9.5)	13.3 (5.1)
*València Bulevard Sud*	97.7 (53.7)	69.7 (51.9)	31.4 (19.2)	19.6 (10.3)	40.8 (21.6)	34.0 (15.9)	39.0 (12.3)	23.1 (8.6)	36.2 (17.2)	25.8 (7.8)	37.8 (16.8)	28.7 (9.8)
*València Pista de Silla*	88.5 (43.9)	75.4 (44.9)	43.3 (21.0)	15.0 (7.2)	47.0 (13.0)	29.7 (11.0)	34.5 (10.4)	27.1 (8.3)	36.2 (12.8)	30.0 (7.2)	34.1 (15.5)	34.1 (11.7)
València Molí del Sol	60.4 (33.4)	36.6 (22.0)	31.2 (14.8)	12.3 (4.8)	30.3 (7.9)	9.6 (4.0)	23.7 (5.6)	10.6 (4.0)	26.5 (9.8)	12.2 (1.5)	24.4 (7.4)	14.7 (6.5)
València Vivers	64.0 (33.9)	56.2 (38.3)	27.1 (15.3)	10.7 (4.5)	26.8 (6.4)	17.0 (5.2)	18.3 (6.7)	19.5 (5.5)	15.8 (7.5)	15.3 (3.0)	19.8 (11.6)	20.7 (8.0)
València Politècnic	50.6 (29.2)	36.7 (25.4)	20.1 (10.9)	8.0 (2.5)	15.8 (4.0)	9.6 (3.2)	11.3 (5.9)	8.6 (2.1)	16.2 (4.1)	8.8 (1.8)	16.5 (9.1)	10.5 (4.0)
**NO_2_**	**Normality 1**		**Lockdown**		**Phase 1**		**Phase 2**		**Phase 3**		**Normality 2**	
	**2019** **µ(σ)**	**2020** **µ(σ)**	**2019** **µ(σ)**	**2020** **µ(σ)**	**2019** **µ(σ)**	**2020** **µ(σ)**	**2019** **µ(σ)**	**2020** **µ(σ)**	**2019** **µ(σ)**	**2020** **µ(σ)**	**2019** **µ(σ)**	**2020** **µ(σ)**
*València Centre*	46.3 (12.3)	33.0 (11.6)	29.0 (9.5)	14.6 (4.7)	28.9 (7.3)	15.8 (5.5)	25.2 (4.0)	16.5 (3.6)	18.8 (2.5)	19.7 (3.1)	30.6 (9.9)	28.0 (7.8)
*València Avd Francia*	39.7 (16.9)	27.1 (10.5)	20.6 (10.0)	6.7 (3.2)	18.8 (4.5)	11.0 (5.2)	12.4 (3.8)	9.4 (3.7)	12.2 (2.6)	7.7 (1.6)	13.9 (7.6)	11.5 (4.1)
*València Bulevard Sud*	51.6 (22.6)	30.4 (13.5)	22.3 (17.0)	14.4 (5.9)	28.8 (6.3)	23.3 (10.5)	26.6 (8.7)	16.9 (6.2)	24.5 (9.5)	17.3 (4.7)	26.3 (11.5)	19.6 (6.2)
*València Pista de Silla*	44.0 (15.9)	34.6 (14.1)	26.7 (11.8)	9.1 (4.5)	28.4 (5.7)	19.8 (7.3)	21.9 (6.2)	17.3 (5.5)	21.3 (5.7)	18.2 (4.4)	21.0 (9.7)	21.0 (6.7)
València Molí del Sol	33.4 (14.3)	18.9 (7.9)	21.4 (9.6)	6.9 (2.9)	20.5 (4.7)	6.5 (3.0)	16.1 (3.7)	6.4 (2.7)	17.3 (5.9)	7.0 (1.1)	16.1 (5.4)	11.2 (5.1)
València Vivers	35.9 (14.1)	33.2 (13.8)	18.4 (9.6)	7.1 (3.2)	20.2 (4.0)	11.9 (4.2)	13.9 (5.2)	14.7 (3.9)	12.0 (4.59	11.5 (1.9)	15.3 (8.79	15.4 (6.0)
València Politècnic	32.2 (14.1)	21.7 (9.9)	14.9 (8.1)	5.3 (1.9)	11.2 (2.7)	7.9 (2.6)	7.8 (5.0)	7.3 (1.9)	12.2 (2.8)	7.0 (1.4)	13.2 (7.6)	8.5 (3.2)
**NO**	**Normality 1**		**Lockdown**		**Phase 1**		**Phase 2**		**Phase 3**		**Normality 2**	
	**2019** **µ(σ)**	**2020** **µ(σ)**	**2019** **µ(σ)**	**2020** **µ(σ)**	**2019** **µ(σ)**	**2020** **µ(σ)**	**2019** **µ(σ)**	**2020** **µ(σ)**	**2019** **µ(σ)**	**2020** **µ(σ)**	**2019** **µ(σ)**	**2020** **µ(σ)**
*València Centre*	31.3 (16.8)	26.6 (15.3)	13.8 (6.6)	7.4 (2.6)	13.1 (4.5)	6.4 (1.2)	11.3 (2.6)	6.9 (1.1)	8.8 (2.1)	7.2 (0.8)	13.2 (4.9)	10.2 (3.1)
*València Avd Francia*	13.8 (13.0)	8.6 (10.0)	3.4 (2.2)	1.9 (1.1)	3.6 (2.1)	2.6 (0.9)	2.5 (0.7)	2.1 (0.4)	2.7 (1.3)	2.0 (0.0)	2.8 (1.7)	1.6 (0.9)
*València Bulevard Sud*	30.4 (23.7)	25.8 (26.4)	6.2 (4.0)	4.0 (3.3)	7.8 (5.3)	7.2 (3.9)	7.9 (3.2)	4.1 (1.8)	7.5 (3.9)	5.5 (2.6)	7.6 (3.8)	6.0 (2.9)
*València Pista de Silla*	29.2 (19.5)	26.7 (21.1)	11.0 (6.7)	4.0 (2.0)	12.1 (5.3)	6.7 (2.4)	8.6 (3.1)	6.4 (1.9)	10.0 (4.8)	7.8 (1.8)	8.7 (4.3)	8.4 (3.6)
València Molí del Sol	17.8 (13.6)	11.6 (10.0)	6.4 (4.0)	3.4 (1.4)	6.4 (2.5)	2.3 (1.1)	5.0 (1.7)	2.8 (1.1)	6.2 (2.6)	3.3 (0.5)	5.5 (1.8)	2.5 (1.3)
València Vivers	18.5 (14.3)	15.2 (17.4)	5.7 (4.1)	2.7 (1.0)	4.5 (2.3)	3.4 (0.9)	3.0 (1.5)	3.2 (0.9)	3.0 (2.1)	2.7 (0.5)	3.1 (2.2)	3.6 (1.4)
València Politècnic	12.3 (10.8)	9.9 (10.9)	3.6 (2.4)	2.1 (0.7)	3.1 (1.4)	1.6 (0.6)	2.7 (0.8)	1.2 (0.4)	2.7 (0.8)	1.5 (0.5)	2.3 (1.5)	1.5 (0.7)

**Table 4:** O_3_ mean and standard deviation are expressed in μg∙m^−3^. A green color denotes significant (*p*-value < 0.05) decreases, whereas an orange color denotes significant increases between 2019 and 2020.

O_3_	Normality 1		Lockdown			Phase 1		Phase 2		Phase 3		Normality 2	
	2019 µ(σ)	2020 µ(σ)	2019 µ(σ)		2020 µ(σ)	2019 µ(σ)	2020 µ(σ)	2019 µ(σ)	2020 µ(σ)	2019 µ(σ)	2020 µ(σ)	2019 µ(σ)	2020 µ(σ)
*València Pista de Silla*	36.1 (17.0)	27.2 (11.8)	67.2 (14.7)	63.5 (12.7)	52.9 (8.2)	77.2 (13.3)	63.0 (5.4)	71.6 (9.3)		63.0 (8.4)	62.5 (6.0)	58.4 (13.1)	62.4 (10.6)
València Molí del Sol	38.4 (17.2)	30.4 (11.2)	69.0 (15.2)	59.7 (10.7)	58.4 (7.2)	68.8 (8.8)	69.1 (4.2)	63.2 (8.5)		67.8 (10.9)	59.0 (3.9)	57.3 (9.7)	57.5 (7.3)
València Vivers	40.5 (16.3)	29.3 (11.8)	73.2 (16.6)	63.9 (9.7)	63.4 (8.4)	73.2 (7.9)	75.9 (4.7)	63.9 (9.3)		78.3 (8.4)	59.7 (4.5)	66.2 (11.4)	63.9 (10.2)
València Politècnic	42.0 (15.2)	32.3 (12.9)	76.9 (15.9)	60.5 (9.4)	67.4 (8.1)	72.3 (11.0)	76.7 (4.9)	69.9 (8.9)		75.5 (8.5)	64.2 (3.7)	63.1 (12.3)	62.2 (8.7)

## Data Availability

Not applicable.
